# Small, Smaller, Nano: New Applications for Potato Virus X in Nanotechnology

**DOI:** 10.3389/fpls.2019.00158

**Published:** 2019-02-19

**Authors:** Juliane Röder, Christina Dickmeis, Ulrich Commandeur

**Affiliations:** Institute for Molecular Biotechnology, RWTH Aachen University, Aachen, Germany

**Keywords:** plant virus, genetic engineering, chemical conjugation, nanoparticles, imaging, drug delivery, bioinspired materials

## Abstract

Nanotechnology is an expanding interdisciplinary field concerning the development and application of nanostructured materials derived from inorganic compounds or organic polymers and peptides. Among these latter materials, proteinaceous plant virus nanoparticles have emerged as a key platform for the introduction of tailored functionalities by genetic engineering and conjugation chemistry. Tobacco mosaic virus and Cowpea mosaic virus have already been developed for bioimaging, vaccination and electronics applications, but the flexible and filamentous Potato virus X (PVX) has received comparatively little attention. The filamentous structure of PVX particles allows them to carry large payloads, which are advantageous for applications such as biomedical imaging in which multi-functional scaffolds with a high aspect ratio are required. In this context, PVX achieves superior tumor homing and retention properties compared to spherical nanoparticles. Because PVX is a protein-based nanoparticle, its unique functional properties are combined with enhanced biocompatibility, making it much more suitable for biomedical applications than synthetic nanomaterials. Moreover, PVX nanoparticles have very low toxicity *in vivo*, and superior pharmacokinetic profiles. This review focuses on the production of PVX nanoparticles engineered using chemical and/or biological techniques, and describes current and future opportunities and challenges for the application of PVX nanoparticles in medicine, diagnostics, materials science, and biocatalysis.

## What A Wonderful World: Plant Virus Nanoparticles

In the rapidly evolving interdisciplinary field of nanotechnology, VNPs are receiving more and more attention due to their outstanding structural characteristics and ease of functionalization compared to synthetic nanoparticles. Plant VNPs are particularly attractive because they are non-infectious in humans and thus inherently safe. Numerous copies of one or more identical CP subunits self-assemble into a defined spherical or rod-shaped particle (depending on the virus species), many of which have been characterized to atomic resolution (Lin et al., 1999; Clare and Orlova, 2010; Lee et al., 2012; Kendall et al., 2013; Agirrezabala et al., 2015). Although different viruses have distinct surface properties, these can easily be tailored to achieve a desired function by genetic engineering or chemical conjugation, or a combination of both, allowing the precise nanoscale control of VNP structure and function. Large quantities of plant VNPs can be produced in the laboratory by molecular farming, in which plants are used as a virus production factory. The resulting VNPs are highly stable under a wide range of conditions.

The two most popular plant VNP platforms are Cowpea mosaic virus and TMV, and their applications have been extensively reviewed (McCormick and Palmer, 2008; Soto and Ratna, 2010; Pokorski and Steinmetz, 2011; Alonso et al., 2013; Love et al., 2014; Wen and Steinmetz, 2016). In contrast, the development of PVX for pharmaceutical and imaging applications has only been discussed in a single review article thus far, even though PVX-based VNPs are unique in their ability to offer multi-functional flexible scaffolds with a high aspect ratio (Lico et al., 2015). In this article, we therefore focus exclusively on the modification of PVX and its applications in medicine, diagnostics, materials science and biocatalysis.

## Come as You Are: Potato Virus X

### The Way I Tend to Be: Characteristics

Potato virus X belongs to the family *Alphaflexiviridae* and is the type member of the genus *Potexvirus* (Adams et al., 2004). It is considered important among plant pathogens that infect agricultural plants of the family *Solanaceae*, especially potato, tomato and tobacco, and is transmitted via mechanical contact.

Potato virus X has a 6.4-kb positive-stranded RNA genome containing five ORFs, with a 5′-methylguanosine cap and a polyadenylated 3′-end (Koonin, 1991; Kim and Hemenway, 1997). The first ORF encodes the 166-kDa RNA-dependent RNA polymerase which is required for virus replication, whereas cell-to-cell movement is mediated by p25, p12 and p8, the products of three overlapping ORFs known as the TGB (Angell et al., 1996; Verchot et al., 1998; Draghici et al., 2009). In addition, p25 is also a silencing inhibitor (Bayne et al., 2005; Chiu et al., 2010). The fifth ORF encodes the CP, multiple copies of which assemble to form the capsid around the genomic RNA. The CP is also important for cell-to-cell and long-distance (systemic) transport through the plant (Tollin and Wilson, 1988; Chapman et al., 1992; Fedorkin et al., 2001; Betti et al., 2012). The virus proteins are translated from three sgRNAs: sgRNA1 (2.1 kb) expresses TGB p25; sgRNA2 (1.4 kb) expresses TGB p12 and p8, the latter by leaky scanning (Dolja et al., 1987; Verchot et al., 1998); and sgRNA3 (0.9 kb) expresses the CP (Dolja et al., 1987).

**FIGURE 1:**
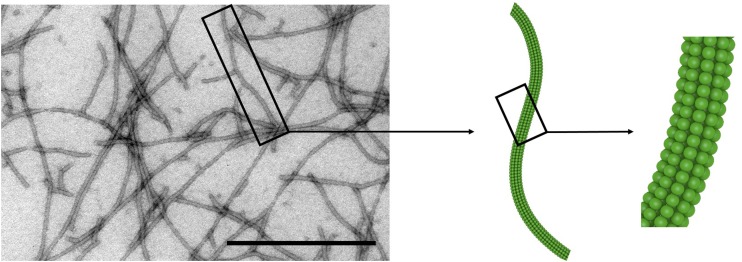
Potato virus X particles. Transmission electron micrograph and schematic representation of PVX. Scale bar = 500 nm. Adapted from Lauria et al. (2017).

The high-resolution structure of isolated PVX CP subunits has not yet been solved, although models have been proposed (Nemykh et al., 2008; Kendall et al., 2013). The 515 × 14.5 nm flexuous rod-shaped particle (Figure 1) comprises 1270 CP subunits with 8.90 ± 0.01 subunits per turn, forming a 3.45 nm helical pitch (Tollin et al., 1980; Parker et al., 2002). Each CP subunit is thought to contain seven α-helices and six β-strands, with the C-terminus located inside the assembled particle and the N-terminus projected externally (Sober et al., 1988; Baratova et al., 1992; Nemykh et al., 2008). The N-terminus therefore provides an excellent site for the presentation of recombinant peptides, which is achieved by introducing the corresponding sequence at the 5′-end of the *cp* gene (see further discussion in Section “Change the World: Genetic Engineering”).

In-frame deletions of the *cp* 5′-end that affect PVX infectivity and particle morphology were first described by Chapman et al. (1992). These deletions were shown to produce intact virions capable of systemic infection, but electron microscopy revealed an atypical twisted morphology similar to that of particles exposed to trypsin (Tremaine and Agrawal, 1972). These data suggested that the N-terminus influences intramolecular and/or intermolecular interactions that stabilize the virus structure.

The apparent molecular weight of the PVX CP as determined by sodium dodecylsulfate polyacrylamide gel electrophoresis changes when the CP is exposed to trypsin, which removes an N-terminal segment. The latter comprises a highly conserved cluster of serine and threonine residues representing potential glycosylation sites. When these are replaced with alanine or glycine the glycans are not added (Kozlovsky et al., 2003; Baratova et al., 2004). The PVX CP contains a single *O*-linked hexose monosaccharide (galactose or fucose) joined to the acetylated serine residues (NAcSer1). These carbohydrates alter the electrophoretic mobility of the CP and induce the formation of a columnar shell of bound water molecules (Tozzini et al., 1994; Kozlovsky et al., 2003; Baratova et al., 2004). Moreover, sequence analysis revealed in-frame deletions affecting the first 29 residues of the CP (Δ29CP) in late infection passages of recombinant PVX particles displaying two Beet necrotic yellow vein virus epitopes (Uhde-Holzem et al., 2007). The N-terminus of the CP is therefore thought to help maintain the helical structure of the virus during assembly, and may influence the assembly and stability of PVX particles. These phenomena must be taken into consideration when creating PVX mutants (see further discussion in Section “Change the World: Genetic Engineering”).

### All the Small Things: Virus Assembly and Virus-Like Particles

In contrast to the *Potexvirus* Papaya mosaic virus (Erickson and Bancroft, 1978), PVX CP subunits have not yet been shown to assemble into filamentous virus-like particles in the absence of RNA either *in vivo* or *in vitro*. This is likely to reflect the specific recognition of the virus genomic RNA by the CP, which plays a key role during the assembly of the virion (Kwon et al., 2005). The genomic RNA region which interacts specifically with the CP is known as the origin of assembly and similar structures have been identified in other plant viruses, e.g., TMV, Brome mosaic virus and Turnip crinkle virus (Butler, 1984; Sit et al., 1994; Miller et al., 1998; Choi and Rao, 2003; Arkhipenko et al., 2011). In the case of PVX, the origin of assembly is at the end of the 5′-region of the RNA, defined as stem loop 1 (Miller et al., 1998; Cho et al., 2012). This secondary structure forms within the nucleotide sequence spanning positions 32–106 of the 5′-region, and consists of four stems (SA, SB, SC, SD), three internal asymmetric loops (LA, LB, LC), and a terminal tetraloop (Park et al., 2008). A portion of the stem loop 1 region comprising nucleotides 32–47 and 86–106 (SA, SB, LA, LB) is likely to adopt multiple conformations (Miller et al., 1998). This secondary structure contributes to functions such as virus replication, translation and cell-to-cell transport, as well as influencing the virion composition (Kim and Hemenway, 1997; Kwon et al., 2005; Lough et al., 2006). It is likely that the functional properties of the origin of assembly are conferred by its structure rather than a particular nucleotide sequence, and this is important for the assembly of PVX with (heterologous) RNAs into VNPs (Kwon et al., 2005; Park et al., 2008; Arkhipenko et al., 2011).

When heated to 70°C, filamentous PVX particles begin to swell at one or both ends (Nikitin et al., 2016). Increasing the temperature to 90°C for 10 s resulted in the formation of spherical PVX virus-like particles (Nikitin et al., 2016), similar to TMV structures formed at higher temperatures, as reported by Atabekov et al. (2011). The average diameter of these spherical PVX particles was 48 and 77 nm at concentrations of 0.1 and 1.0 mg ml^-1^, respectively. However, increasing the virus concentration to 10 mg ml^-1^ did not cause any further change in the diameter. These particles did not contain RNA and were not resistant to detergents such as 0.15% sodium dodecylsulfate. Analysis of the predicted secondary structure of the denatured CP revealed some differences compared to the filamentous PVX particles (DiMaio et al., 2015). The α-helical content was 14–19% and the β-sheet content was 28–99%, with 53% of the protein remaining unordered. However, most native epitopes were retained on the particle surface so these atypical particles may still be suitable for the presentation of antigens.

### Don’t Stop ‘Til You Get Enough: Production of VNPs

PVX VNPs are typically produced by the infection of tobacco (*Nicotiana*) species, including *N. benthamiana, N. tabacum* Xanthi nc or Samsun NN, *N. clevelandii* or *N. glutinosa*. To propagate the (recombinant) virus, plants are inoculated with a PVX-derived vector comprising a cDNA copy of the viral genome under control of the Cauliflower mosaic virus 35S promoter. The vector contains either the wild-type *cp* gene or a corresponding gene fusion allowing the external display of a peptide. Plants can also be infected with the cDNA copy using *Agrobacterium tumefaciens*. However, this approach is mostly used to express recombinant proteins from deconstructed PVX vectors rather than the production of VNPs (Peyret and Lomonossoff, 2015). The surface of 4-week-old plants is gently treated with Celite 545 or a similar abrasive and three leaves are inoculated with 10 μg plasmid DNA (Lee et al., 2014). Infected plants should be harvested 14–21 dpi, which is a sufficient time for the establishment of a systemic infection. Particles are usually purified according to a modified protocol from the International Potato Center (Lima, Peru). Detailed protocols have been published (Lee et al., 2014; Lauria et al., 2017; Shukla et al., 2018).

Once successful infection is established, the VNPs can easily be propagated by using plant extracts or purified VNPs for the direct inoculation of uninfected plants (Uhde-Holzem et al., 2007). However, the genetic instability of the recombinant RNA genomes over serial passages of infection is a major limitation (Avesani et al., 2007; Dickmeis et al., 2014). Vector DNA or plant extracts from the first round of infection should therefore be used to ensure the reproducible production of VNPs.

## Master of Puppets: Strategies for the Creation of Modified PVX Nanoparticles

**FIGURE 2:**
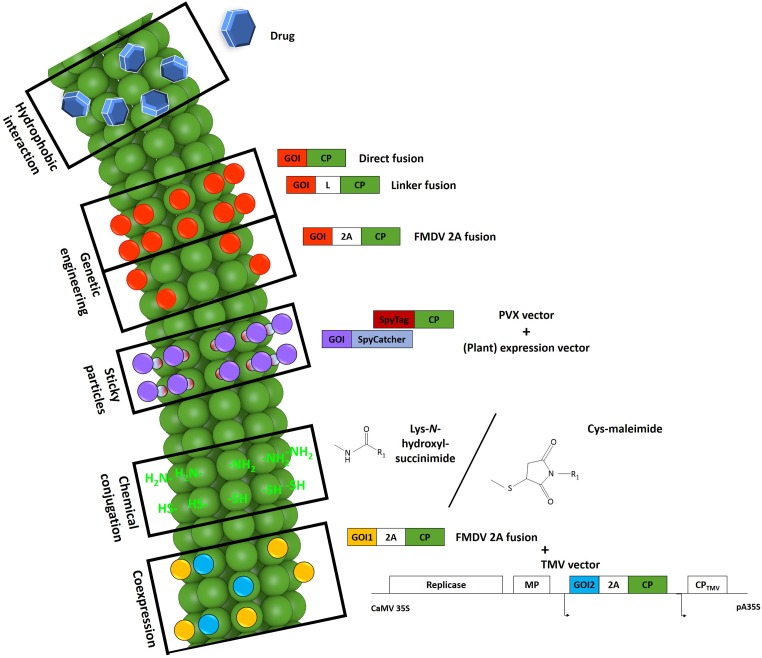
Schematic representation of modifications evaluated for the production of PVX-based VNPs. Certain drugs (blue hexagons) such as doxorubicin can intercalate between the coat protein (CP) subunits of particles via hydrophobic interactions (Le et al., 2017b; reproduced by permission of The Royal Society of Chemistry). By genetic engineering, it is possible to fuse a target sequence to the 5′-end of the *cp* gene as a direct fusion, with an additional linker, or with an intervening 2A sequence. This strategy leads to the production of particles carrying the protein of interest (red) on every CP copy as direct fusions or on ∼25% of the CP copies with the 2A sequence. The fusion of a SpyTag peptide (dark red) to the particle surface makes it possible to attach any protein of interest (purple) fused to a SpyCatcher protein (light blue) expressed in any system. The PVX particle surface also features reactive groups (bright green) offered by lysine and cysteine residues that can be used for the chemical coupling of target molecules (R_1_). *N*-hydroxysuccinimide or maleimide chemistry is typically used for this purpose. For the successful presentation of several large proteins utilizing the 2A sequence (yellow), a second set of a PVX CP fusion constructs (blue) can be co-expressed in the same cell by co-infection with a TMV vector. 2A, Foot-and-mouth disease virus 2A sequence. CaMV 35S/pA35S, Cauliflower mosaic virus 35S promoter/polyadenylation sequence. CP, coat protein. GOI, gene of interest. L, linker. MP, movement protein. Small arrows indicate subgenomic promoter-like sequences. Adapted from Le et al. (2017b).

Modified PVX nanoparticles can be produced by methods that result in either a permanent or reversible functionalization introduced by genetic engineering and/or chemical conjugation. The choice of production approach depends on which properties are required in the VNP. Thus far, PVX nanoparticles have been used as scaffolds for external peptide presentation. Given that particles can only form in the presence of genomic RNA, the steric limitations of the virus morphology make it difficult to display peptides in the internal channel or to use this channel to carry a payload of drugs or imaging molecules. Exceptionally, hydrophobic substances such as the drug doxorubicin have been used for traceless deposition by spontaneous attachment to the surface grooves of the virus (Le et al., 2017b). However, the major drawback of this method is the need for a high molar excess of the drug and a long reaction time. Figure 2 summarizes the types of modifications used thus far for the production of PVX-based VNPs.

### Change the World: Genetic Engineering

Genetic engineering is the preferred strategy to modify VNPs when the aim is to display single amino acids or small peptides. Recombinant PVX particles can be created by adding the target sequence in frame at the 5′-end of the *cp* gene, whereas insertions at the 3′-end tend to be detrimental, probably because they inhibit virus replication and assembly (Chapman et al., 1992; Hoffmeisterova et al., 2012). Target sequences introduced at the 5′-end must meet certain criteria to allow the virus to assemble into functional particles and move locally and systemically within the plant. A major limitation in this context is the size of the target peptide, which generally must be no longer than 60 amino acids (Uhde-Holzem et al., 2016). This is because PVX avoids extra genetic load by selecting against larger insertions. However, we recently found that the fluorescent protein iLOV (113 amino acids) could be fused directly to the CP without impairing the assembly and systemic movement of the virus (Röder et al., 2018) as discussed further in Section “Ligth Me Up: Imaging With PVX VNPs”. Virion assembly is sensitive to steric hindrance (Dawson et al., 1989; Cruz et al., 1996), and cell-to-cell movement is inhibited by the presence of too many tryptophan residues (Lico et al., 2006). Furthermore, the absence of serine and threonine (Lico et al., 2006; Betti et al., 2012) or the presence of too many positively charged amino acids (Uhde-Holzem et al., 2007) can make the virus unstable resulting over several serial passages in the selection of compensatory deletion mutants. In fact, serine and threonine residues are essential for phosphorylation and glycosylation thereby stabilizing the particles by creating a surrounding water shell (Baratova et al., 2004; Lico et al., 2006). The pI is another factor to consider when designing a CP fusion protein. If the pI of the CP fusion is in the range 5.2–9.2, the assembled particle can move systemically (Lico et al., 2006; Uhde-Holzem et al., 2007). Otherwise, the pI must be adjusted by introducing a compensatory sequence such as the acidic DEADDAED peptide (Röder et al., 2017). Certain peptides favor an additional flexible glycine/serine-rich linker, including mineralization-inducing peptide 3 (MIP3) as discussed in Section “Material Girl: PVX for Biomaterial Applications” (Lauria et al., 2017).

The assembly of particles comprising CP fusion proteins containing more than 60 additional amino acids can be facilitated by mixing recombinant and wild-type CPs, thus overcoming the steric hindrance between recombinant CPs in homogeneous recombinant particles. This can be achieved by introducing the ribosomal skip sequence from Foot-and-mouth disease virus (FMDV), known as the 2A sequence, between the 3′-end of the inserted sequence and the 5′-end of the wild-type *cp* gene (Cruz et al., 1996; Donnelly et al., 2001). This overcoat strategy allows entire proteins to be presented on recombinant virus particles, including fluorescent proteins (Cruz et al., 1996; Shukla et al., 2014a), enzymes such as lipase (Carette et al., 2007), epitopes (Marconi et al., 2006; Zelada et al., 2006; Uhde-Holzem et al., 2010), the rotavirus VP6 protein (O’Brien et al., 2000), and a single-chain antibody (Smolenska et al., 1998), as discussed further in Section “Knowing Me, Knowing You: Biosensing.” However, the 2A sequence does not ameliorate the general instability of vectors carrying large inserts, and selection pressure still tends to favor their deletion (Scholthof et al., 1996). For example, the GFP sequence was deleted from the vector PVX-GFP-2A-CP after 28 days (Shukla et al., 2014a).

Vaculik et al. (2015a) identified four promising internal transgene insertion positions within the surface loops of the PVX CP. The tested epitope insertion mutant was infectious and produced particles only after amino acid 23 (Vaculik et al., 2015b). However this position still belongs to the N-terminal intrinsically disordered domains of *Potexviruses* (Solovyev and Makarov, 2016) and is therefore not essential for particle assembly. This was proven by Chapman et al. (1992) by removing codons 7–31 of the PVX CP leading to virions with atypical morphology. In fact, spontaneous deletions in this region occur during infection with recombinant particles displaying epitopes (Lico et al., 2006; Uhde-Holzem et al., 2007). Furthermore deletions up to residue 29 successfully produced particles though only in low amounts (Lico et al., 2006; Uhde-Holzem et al., 2007; Dickmeis et al., 2014). An epitope insertion immediately after amino acid 23 might therefore be beneficial for virus stability and yield.

### Catch Me If You Can: Sticky Particles

In addition to the limitations conferred by the size constraints and instability of inserted sequences (see Change the World: Genetic Engineering), another issue is the inability to present large peptides and proteins that require posttranslational modifications for functionality, because virus replication and assembly occurs exclusively in the cytoplasm. This problem can be addressed using chemical conjugation methods, as demonstrated by the conjugation of the heavy chain of the breast cancer drug Trastuzumab/Herceptin (Esfandiari et al., 2016). However, chemical methods for the attachment of proteins require a 1000-fold molar excess of the protein and very long reaction times, yet still result in poor conjugation efficiencies (typically of 21–86%) depending on the conjugation strategy and the size of the target molecule (Schlick et al., 2005; Holder et al., 2010; Venter et al., 2011; Wen et al., 2012). These cases clearly reveal the need for a rapid and site-specific covalent immobilization method. We recently demonstrated the stable attachment of a functional endoglucanase to PVX using the SpyTag/SpyCatcher (ST/SC) system (Zakeri et al., 2012; Röder et al., 2017). We modified PVX VNPs to display the short ST (see A Little More Action, Please: Catalysis), allowing the rapid and specific irreversible attachment of a SC fusion protein with, in this case, a ∼70% coupling efficiency. Problems resulting from chemical coupling or genetic engineering methods, including size constraints and inappropriate amino acid compositions, can be overcome using this approach. PVX-ST VNPs therefore provide a universally applicable platform with great promise for future practice.

### The Chemistry Between Us: Chemical Addressability

Potato virus X can be modified not only by genetic engineering but also by chemical conjugation, which is advantageous when the functionalization is conferred not by small peptides but by whole proteins, polymers or small molecules such as fluorescent dyes. Each PVX CP bears numerous amine and carboxylate groups among its 11 lysine, 10 aspartic acid, 10 glutamic acid and 3 cysteine residues, although only a single lysine residue and a single cysteine residue are exposed to the solvent, making them addressable using *N*-hydroxysuccinimide and maleimide chemistry, respectively (Pierpoint, 1974; Gres et al., 2012; Le et al., 2017a). PVX can be made more amenable to conjugation reactions by inserting additional amino acids carrying suitable exposed side chains, using the genetic engineering methods described in Section “Change the World: Genetic Engineering” (Wang et al., 2002; Geiger et al., 2013). Further potential targets for chemical modification are the glycans present in some strains of PVX, but earlier studies showed that they are not addressable (Gres et al., 2012). Various conjugation methods including click chemistry have been comprehensively reviewed (Pokorski and Steinmetz, 2011). However, these methods suffer from poor conjugation efficiencies and a large molar excess of the target molecule is generally required (Schlick et al., 2005; Holder et al., 2010; Venter et al., 2011). The fluorescent dye OregonGreen 488 was conjugated to PVX particles using both Lys-*N*-hydroxysuccinimide and Cys-maleimide chemistry, with the former achieving the best performance resulting in the modification of up to 15% of the CPs (Le et al., 2017a). This may reflect the low accessibility of the Cys residue, which is thought to be located within a surface groove.

### Two Princes: PVX Coat Protein Expression Using a TMV Co-vector

For the construction of peptide vaccines, it is often advantageous to present several different epitopes on a single scaffold to induce a strong immune response (Sette and Peters, 2007). This can be challenging when using plant viruses because viruses of the same species with different CP modifications cannot achieve simultaneous infections due to the phenomenon of super-infection exclusion (Folimonova, 2012; Julve et al., 2013; Zhang et al., 2018). The presentation of several epitopes on a plant virus can be achieved by (separate) heterologous expression followed by *in vitro* assembly (Eiben et al., 2014; Tyler et al., 2014; Jin et al., 2016). However, as stated in Section “All the Small Things: Virus Assembly and Virus-Like Particles”, PVX cannot assemble without its genomic RNA and *in vitro* assembly does not achieve high yields of VNPs.

To address these issues, we developed an expression system for the construction of chimeric PVX particles displaying different proteins as CP fusions. We used combinations of PVX and TMV expression vectors each expressing different PVX CP fusions and achieved proof of principle using fusion proteins containing GFP and mCherry as well as split-mCherry (Dickmeis et al., 2015). We also reported the first co-expression of CP_PVX_ using a full-sized PVX expression vector, a remarkable achievement given that the expression of a virus CP often leads to cross-protection against other strains of the same virus (Gal-On and Shiboleth, 2006; Lin et al., 2007). The expression of CP_TMV_ by PVX prevents co-infection with a TMV vector, leading to CP-mediated resistance in *N. benthamiana* (Lu et al., 1998). In contrast, the expression of CP_PVX_ by TMV does not have this effect, allowing robust co-expression with the PVX vector. The TMV-derived CP_PVX_ did not appear to inhibit PVX infection although the TMV infection process is faster and CP_PVX_ is expressed in the cells before the PVX vector gains entry. In contrast, we found that PVX infection was enhanced by the TMV vector, yielding brighter fluorescence for the PVX-expressed fluorescent proteins. The enhancement of PVX in TMV/PVX co-expression systems is well known (Goodman and Ross, 1974) and was also observed in our combination, whereas no CP-mediated resistance against PVX was detected. Our system was therefore able to achieve the co-presentation of GFP and mCherry on PVX particles as well as the reconstruction of split-mCherry on the particle surface.

## Learn to Fly: Applications (Figure 3)

**FIGURE 3:**
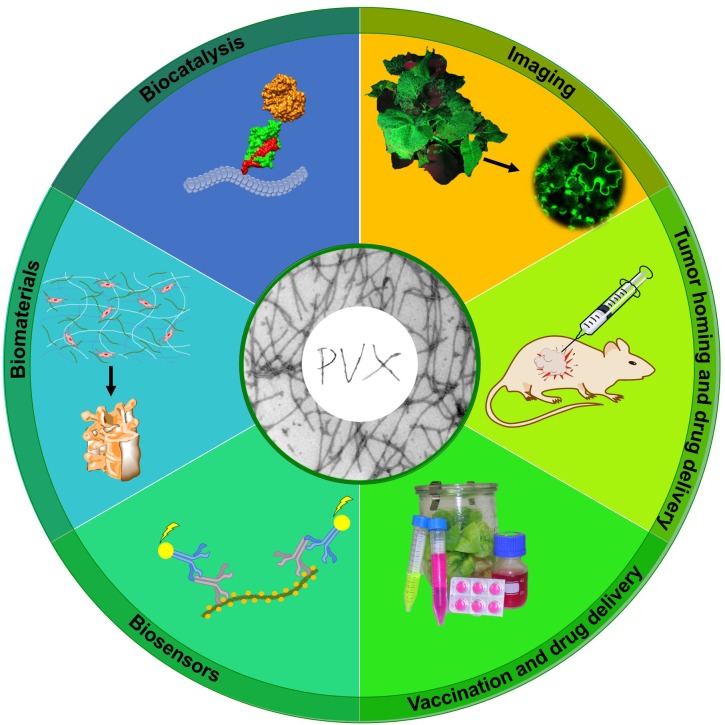
Applications of PVX nanoparticles. PVX has been evaluated for diverse applications including molecular imaging, tumor homing and drug delivery, vaccination, biosensor design, biomaterials development, and biocatalysis. Adapted from Röder et al. (2017).

### What Will Become of Us: *In vivo* Fate and Cytotoxicity of PVX Nanoparticles

Virus nanoparticles have many advantages as carrier systems for drugs and imaging molecules, but their repetitive proteinaceous structures (reflecting the assembly of multiple identical CPs) can induce an immune response, which is a barrier to clinical translation (Lico et al., 2009). The ordered and multivalent structures formed by both helical and icosahedral capsids also appear to function as pathogen-associated molecular patterns, which can be recognized by the innate immune system and elicit robust cellular and humoral immune responses (Lizotte et al., 2015). Thus, VNPs intended for medical applications must be evaluated for risks, and their fate and potential immunogenic or cytotoxic effects must be determined.

The bioavailability of VNPs *in vivo* can be controlled by modifying their surface chemistry, and the introduction of targeting ligands can facilitate their interactions with specific cell types. For example, VNPs carrying ligands recognized by receptors on cancer cells can be used to deliver toxic drug payloads to tumors, resulting in the accumulation of drugs inside the tumor while reducing systemic side effects. In addition to these active targeting mechanisms, the accumulation of nanoparticles in tumors also occurs via passive processes, which are enhanced by the higher aspect ratio of PVX (see Killing in the Name: Tumor Homing and Drug Delivery). Nanoparticle-mediated drug delivery can be achieved using lipid-based micelles, carbon nanotubes, metal nanoparticles, polymeric capsules, iron oxide nanoparticles, or protein-based particles and nanocages (Bhattacharya et al., 2014; Jin et al., 2018; Khan et al., 2018; Singh et al., 2018; Vallabani and Singh, 2018; Xiao et al., 2019). The shape of the nanoparticles has a huge impact on their *in vivo* behavior, particularly tissue accumulation and clearance from the circulatory system (Lee et al., 2015). The filamentous shape of PVX offers several advantages in this context because elongated materials evade the immune system more effectively, reducing the quantity of particles lost by macrophage uptake (Arnida et al., 2011; Vácha et al., 2011). Furthermore, coating VNPs with the uncharged, hydrophilic polymer PEG can also reduce their immunogenicity by preventing undesirable non-specific cell interactions, thus prolonging the plasma circulation time and increasing their stability (Lewis et al., 2006; Steinmetz and Manchester, 2009; Shukla et al., 2013). Many functionalized PEG monomers and chains are available for the modification of nanomaterials (Raja et al., 2003; Bruckman et al., 2014; Lee et al., 2015). PEG coating creates a hydrophilic shield, which inhibits serum protein adsorption and confers stealth properties that increase the circulation time and also reduce the tendency of VNPs to accumulate in the liver and spleen (Owens and Peppas, 2006; Soo Choi et al., 2007; Ruggiero et al., 2010; Huang et al., 2011).

In healthy mice, PEGylated PVX particles accumulate in the white pulp regions of the spleen, and to a lesser extent in the liver and kidneys, 2–6 h after intravenous administration. This indicates that PVX is mainly sequestered by the mononuclear phagocyte system in the spleen and liver (Shukla et al., 2014b). Non-PEGylated PVX particles were also shown to adhere to red blood cells and penetrate the white pulp of the spleen (Lico et al., 2016). PEGylated PVX particles co-localize with F4/80-positive macrophages, probably Kupffer cells, in the liver (Shukla et al., 2014b). Filamentous nanomaterials in the same size range as PVX are usually cleared by the mononuclear phagocyte system, but are not excreted by the renal system (Bartneck et al., 2012; Sa et al., 2012; Raza et al., 2017). However, renal clearance cannot be ruled out given the small dimensions along the PVX short axis, and the potential presence of part-digested or broken VNP fragments. Strong PVX fluorescence signals were also observed in the stools of the injected mice, suggesting that some particles are also cleared through the hepatobiliary system. More detailed analysis revealed the accumulation of PVX in B-cells and a higher number of T-cells in the spleen, which may reflect the immunogenicity of PVX and accordingly the induction of humoral and cellular immune responses (Shukla et al., 2014b). PVX was cleared from tumors and other tissues after 5 days, and the strong fluorescence signal from the kidney indicated PVX degradation followed by renal filtration to the bladder.

Blandino et al. (2015) studied the fate and cytotoxicity of filamentous PVX particles and icosahedral Tomato bushy stunt virus particles in hemolysis assays and early embryo assays. Their data showed that the virus particles were very robust and were still able to infect plants after serum incubation for up to 24 h. The hemolysis assay revealed that 10 μg of PVX particles had no effect on erythrocytes *in vitro*, whereas 100–200 μg caused the slight and dose-dependent induction of hemolysis. However, the rate of hemolysis (1.8–2.7%) was far lower than the 5% threshold mandated for biomaterials under ISO/TR 7406 (Li et al., 2011, 2012) with a very high VNP/erythrocyte ratio (Blandino et al., 2015). The early embryo assay is used to determine the teratogenic potential of substances during the first week of embryonic development (Henshel et al., 2003). PVX showed no signs of toxicity or teratogenicity at doses ranging from 1 to 10 μg per embryo, whereas carbon nanotubes induced up to 50% mortality as well as embryo malformations (Blandino et al., 2015). Furthermore, we observed no evidence of apoptosis when we seeded human mesenchymal stem cells onto a PVX-coated surface (Lauria et al., 2017).

Potato virus X nanoparticles are much safer than mammalian viruses for clinical use because they neither infect nor replicate in mammals (Manchester and Singh, 2006). Plant VNPs at doses of up to 100 mg (10^16^ particles) per kg body weight showed no sign of clinical toxicity, which indicates that high concentrations could be used for the targeted destruction of tumors (Kaiser et al., 2007; Singh et al., 2007).

### Killing in the Name: Tumor Homing and Drug Delivery

Non-spherical materials achieve better tumor homing and margination toward vessel walls than spherical particles (Cai et al., 2007; Geng et al., 2007; Gentile et al., 2008; Christian et al., 2009; Lee et al., 2009; Decuzzi et al., 2010; Magee, 2012) and present ligands more efficiently to target cells as well as the larger and flatter vessel wall (Lee et al., 2009; Doshi et al., 2010; Tan et al., 2013). They also achieve more efficient tumor penetration than spherical particles (Nederman et al., 1983; Netti et al., 2000; Thurber et al., 2008; Stylianopoulos et al., 2010a,b) and positively charged materials (Dellian et al., 2000; Stylianopoulos et al., 2010a,b; Ma and Hidalgo, 2013; He and Pistorius, 2017).

Potato virus X accumulates passively in tumors due to the enhanced permeability and retention effect (Shukla et al., 2013). The tumor homing of PEGylated PVX has been demonstrated in several rodent models, including human tumor xenografts of fibrosarcoma, squamous cell sarcoma, colon cancer, and breast cancer (Shukla et al., 2013, 2014b). Successful delivery requires PVX to enter the tumor microcirculation followed by extravasation into the tumor tissue. Filamentous particles show enhanced penetration behavior and better retention because they are transported across membranes more efficiently (Gentile et al., 2008; Lee et al., 2009; Toy et al., 2011). PEGylated PVX particles injected into mice also accumulate in the liver and spleen because these organs are part of the reticuloendothelial system, which removes proteinaceous antigens from circulation (Peiser et al., 2002).

Potato virus X can be loaded with doxorubicin due to the spontaneous hydrophobic interactions and π-π stacking of the planar drug molecules and polar amino acids. Approximately 850–1000 drug molecules are carried by an unmodified PVX particle, indicating that 70–80% of the CPs become stably attached to the drug (Le et al., 2017b; Lee et al., 2017). Doxorubicin remains cytotoxic when loaded onto PVX but its efficacy is lower than that of the free drug, as previously reported for synthetic nanoparticles (Yoo and Park, 2000) and other VNPs (Ren et al., 2007; Lockney et al., 2011). This reflects the different cellular uptake and processing pathways probably used for nanoparticles and small molecules, with the free drug more likely to enter the cell by diffusion across the cell membrane whereas VNPs are taken up by endocytosis or macropinocytosis. No statistical differences in the tumor growth rate or survival time were observed when PVX formulation was compared to the free drug, but the tumor volume was slightly lower in mice treated with the PVX formulation. Furthermore, the PVX formulation did not improve the treatment but the cytotoxic efficacy was maintained. The PEGylation of PVX increased its ability to carry doxorubicin, allowing the attachment of 1000–1500 drug molecules per particle (Le et al., 2017b). As a topical treatment, such VNPs achieve excellent blood and tissue compatibility (Bruckman et al., 2014; Lee et al., 2015), thus opening the door for possible intravenous, systemic administration.

### Light Me Up: Imaging With PVX VNPs

Plant viruses labeled with fluorescent proteins are often used to follow infections in host plants and to unravel the function of viral proteins (Tilsner and Oparka, 2010; Barón et al., 2016; Folimonova and Tilsner, 2018). PVX can be used as a tool for optical imaging by preparing mCherry and GFP overcoat structures using the FMDV 2A sequence (Shukla et al., 2014a). These particles allow the infection of plants to be visualized clearly (Baulcombe et al., 1995; Shukla et al., 2014a).

**FIGURE 4:**
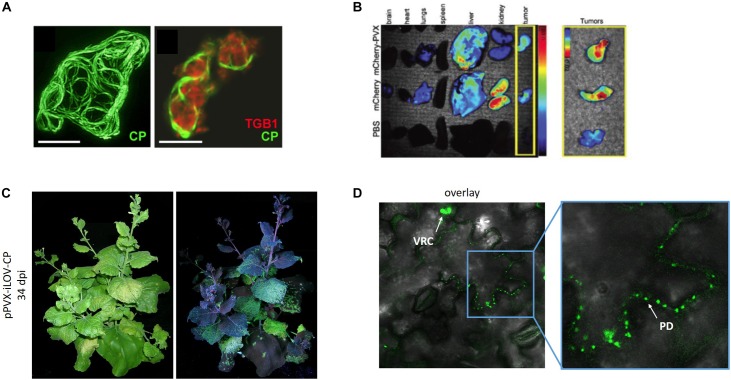
PVX for imaging applications. **(A)** GFP-labeled CP and mCherry-labeled TGBp1 for subcellular localization. Reproduced with permission from Tilsner et al. (2012). Scale bar = 10 μm. **(B)** Biodistribution of PVX-mCherry particles within the organs and tumors of mice (Shukla et al., 2014a). **(C)** Infection of *N. benthamiana* with PVX carrying iLOV and **(D)** localization of iLOV-CP direct fusion proteins within plasmodesmata (Röder et al., 2018). PD, plasmodesmata. VCR, viral replication complex. Adapted from Tilsner et al. (2012), Shukla et al. (2014a), and Röder et al. (2018).

Green fluorescent protein and mCherry have been fused to the TGB proteins and the CP to determine the structure of PVX intracellular replication complexes (Tilsner and Oparka, 2010; Tilsner et al., 2012, 2013; Linnik et al., 2013) (Figure 4A). PVX cell-to-cell movement can usually be observed in leaves 6–10 dpi by the appearance of fluorescent spots, which slowly undergo radial expansion from the inoculation site. When the infected zone reaches the veins, particles are transferred to the vascular bundles enabling long-distance movement. The characteristics of the CP fusion protein can influence the time taken to achieve local and systemic movement. For the fusion protein GFP-2A-CP, fluorescent spots in non-inoculated leaves appear 12–16 dpi (Baulcombe et al., 1995; Shukla et al., 2014a) whereas the smaller iLOV-2A-CP fusion protein spreads more rapidly, with systemic infection appearing as early as 6 dpi (Röder et al., 2018). Later during infection, the virus exclusively spreads from photosynthetically active source tissues to developing sink leaves on the shoot of the plant (Figure 4C). The 2A sequence used in the GFP and mCherry fusion constructs produces a 1:3 ratio of fusion proteins to wild-type CP (Shukla et al., 2014a, 2018). Confocal laser scanning microscopy revealed that the labeled 2A-CP_PVX_ particles were able to move between epidermal cells, as indicated by the presence of a fluorescent signal in the plasmodesmata (Oparka et al., 1996; Cruz et al., 1998; Chapman et al., 2008; Tilsner et al., 2013; Röder et al., 2018) (Figure 4D). One large fluorescent viral replication complex per infected cell is often observed in established infections (Tilsner et al., 2012, 2013; Linnik et al., 2013). These so-called virus factories coordinate the infection processes (Linnik et al., 2013). Additional diffuse fluorescence can be observed in epidermal cells, representing the presence of free fluorescent proteins. This leads to a relatively high background of free fluorescent protein in the cells, preventing the detailed analysis of CP localization. The major disadvantage of the overcoat principle is the unpredictable ratio of fusion protein to wild-type CP_PVX_, but this can be adjusted by using different variants of the FMDV 2A sequence (Luke et al., 2009; Minskaia and Ryan, 2013). Interestingly, we were able to create a direct fusion of the 113-amino-acid residue iLOV protein to the CP that was still able to achieve systemic infection, which is the largest CP fusion reported thus far (Röder et al., 2018). As a tool for the imaging of viral cell-to-cell movement, fluorescent proteins should be densely arrayed on the virus surface to achieve a bright signal, as shown for the iLOV-CP_PVX_ direct fusion (Figure 4D).

Fluorescent VNPs have advantages in other imaging applications compared to inorganic templates such as gold particles and carbon nanotubes because they are biocompatible and do not aggregate under physiological conditions or persist in tissues, which can lead to cell damage (Liu et al., 2008, 2013; Semmler-Behnke et al., 2008; Gad et al., 2012; Jaganathan and Godin, 2012). Filamentous VNPs not only offer a large surface area for the presentation of fluorescent proteins or dyes without quenching (Brunel et al., 2010) but also undergo a two-step clearance process with a plasma circulation half-life of ∼100 min, whereas spherical VNPs have a half-life of 4–7 min (Singh et al., 2007; Bruckman et al., 2014). PVX particles labeled with fluorescent proteins can be produced in and purified from plants and used directly for further applications. For example, we used mCherry-2A-CP PVX nanoparticles to easily determine the biodistribution of PVX in C57BL/6 mice. This revealed that mCherry-PVX is cleared via the reticuloendothelial system and deposited in the liver, resulting in tissue clearance 7 days after administration (see What Will Become of Us: *In vivo* Fate and Cytotoxicity of PVX Nanoparticles). We were also able to show that mCherry-PVX particles are taken up by human HT-29 (colon) tumor cells and localized in the perinuclear region, which was consistent with previous experiments involving PVX labeled with organic dyes (Shukla et al., 2013, 2014a,b, 2015) (Figure 4B). Given that plant VNPs are non-toxic in humans, long-term imaging is also possible. PEG can be used to reduce non-specific interactions and evade the immune system, thereby prolonging circulation times, e.g., for the visualization of blood flow *in vivo* (see What Will Become of Us: *In vivo* Fate and Cytotoxicity of PVX Nanoparticles) (Lewis et al., 2006; Leong et al., 2010; Bruckman et al., 2014; Lee et al., 2015).

### Staying Alive: Vaccination Applications

Plants are considered a promising alternative production system for pharmaceuticals and have been extensively studied for this purpose (Fischer et al., 2012; Lico et al., 2012; Melnik and Stoger, 2013). Plant virus particles or CPs are ideal for the presentation of epitopes (Porta and Lomonossoff, 1998; Yusibov et al., 2006; Rosenthal et al., 2014) and thus can serve as carrier molecules, enhancing the immunogenicity of peptides by presenting them robustly to the immune system (Lomonossoff and Evans, 2014). Immune responses against single pathogen epitopes are in most cases insufficient to provide protection against an infection (Sette et al., 2001; Awram et al., 2002; Sette and Peters, 2007). The presentation of several different epitopes from the same pathogen or numerous different pathogens is ideal for the construction of efficient vaccines. Many pathogens exist as different genotypes or subtypes, for example in the case of Hepatitis C virus (Simmonds et al., 2005). This makes vaccine development more challenging, and is further complicated by the degree of heterogeneity in infected individuals due to the pathogen mutation rate (Hayashi et al., 1999). An advantage of PVX nanoparticles as vaccine candidates is that the presence of the plant virus RNA may trigger Toll-like receptor 7 on antigen-presenting cells, hence boosting the immune response like an adjuvant (Jobsri et al., 2015). Several epitopes have been presented as PVX CP fusions for the production of vaccines (Table 1). The epitope fusions, which were tested in immunization studies, promoted a robust immune response in different animal models. The development of PVX-based vaccines has been comprehensively reviewed (Lico et al., 2015). For the presentation of epitopes on PVX, the major goal is very dense particle coverage by the selected peptides, which favors a strong immune response. Therefore, most of the constructs tested thus far in mice have been direct fusions (Brennan et al., 1999; Marusic et al., 2001; Lico et al., 2009; Cerovska et al., 2012). However, as discussed in Section “Change the World: Genetic Engineering,” not all peptide sequences are suitable for direct fusion to the *cp* and constructs including the 2A sequence, which result in the less-dense presentation of peptides, have been used successfully for the immunization of rabbits (Marconi et al., 2006) and mice (Uhde-Holzem et al., 2010). In one study, the B-cell epitope from the extracellular domain of the human epidermal growth factor receptor 2 was chemically coupled to the CP for presentation on the surface of PVX, followed by the successful immunization of mice (Shukla et al., 2014c, 2017).

**Table 1:** Epitopes presented on PVX-based VNPs for vaccination applications.

Presented epitope	Presentation strategy	Immunization	Reference
D2 peptide from *S. aureus* fibronectin-binding protein	Direct fusion	C57BL/6 mice and rats with adjuvants	Brennan et al., 1999
Linear 2F5 epitope of the Human immunodeficiency virus type 1 glycoprotein gp41	Direct fusion	C57BL/10 mice	Marusic et al., 2001
Major histocompatibility complex class I-restricted peptide of the Influenza A virus nucleoprotein	Direct fusion	C57BL/6 mice with and without adjuvants	Lico et al., 2009
Human papilloma virus 16 E7ggg oncoprotein	Direct fusion	N/D	Plchova et al., 2011
Human papilloma virus 16 L2 minor capsid protein (amino acids 108–120)	Direct fusion	C57BL/6 mice including adjuvants	Cerovska et al., 2012
Rotavirus major inner capsid protein VP6	2A sequence	N/D	O’Brien et al., 2000
Classical swine fever virus E2 glycoprotein	2A sequence	Rabbits with adjuvants	Marconi et al., 2006
*Mycobacterium tuberculosis* ESAT-6 antigen	2A sequence	N/D	Zelada et al., 2006
R9 peptide of the hypervariable region I of Hepatitis C virus	2A sequence	BALB/c mice	Uhde-Holzem et al., 2010
B-cell epitope from the extracellular domain of HER2	Chemical coupling	FVB/N mice with adjuvants	Shukla et al., 2014c


### Material Girl: PVX for Biomaterial Applications

Potato virus X is also promising as a building block for hybrid organic–inorganic materials. Inspired by natural protein-based biomineralization systems such as silaffins (Kröger et al., 1999; Foo et al., 2004), we used PVX as a means to induce the deposition of silica, which could allow the development of new biomaterials with combined surface properties. Silica deposition on templates often involves the use of alkoxysilane precursors such as tetraethyl orthosilane, tetramethyl orthosilane or (3-aminopropyl)triethoxysilane. Genetically modified PVX particles presenting the amino acid sequence YSDQPTQSSQRP fused to the N-terminus of the CP were able to promote mineralization with tetraethyl orthosilane at room temperature, allowing the development of hybrid materials with two or even three components designed using immunogold labeling (Van Rijn et al., 2015). Several VNPs have been shown to arrange themselves around a central core of mesoporous silicon dioxide, extending the virus–silica morphology up to 1–2 μm in diameter and forming higher-order structures. Drygin et al. (2013) reported the selective electroless deposition of platinum ions on one end of PVX particles with nucleation centers 1–2 nm in diameter, although the reason for the unipolar deposition remains unexplained.

Plant viruses also offer new solutions for the biomedical application of biomaterials. For example, biomimetic nanocomposites for the replacement and regeneration of defective bone tissue must achieve biocompatibility while promoting cell adhesion and proliferation. Biological interactions between the implanted biomaterial and the surrounding tissue can only occur if the appropriate physical and cellular signals are present. In a natural context, MIPs are required for the hydroxyapatite mineralization of collagen and they control apatite nucleation and growth (Fisher et al., 2001; George and Veis, 2008). These non-collagenous proteins in the dentin extracellular matrix mainly consist of polar and charged amino acids. PVX displaying similar peptide sequences was able to attract calcium phosphate derivatives when incubated in hydroxyapatite or simulated body fluid (Lauria et al., 2017). Small nucleation centers formed along the longitudinal axis of the particles. Due to the unique atomic precision of the particle assembly, some aspects of the extracellular matrix were mimicked, including the mineral phase of human bone. Hydrogels are widely used as biocompatible scaffolds for tissue engineering, but often lack the signals required for cell interactions (Lee et al., 2016; Naahidi et al., 2017). Therefore, PVX was engineered to display an arginine, glycine and aspartic acid (RGD) peptide, a fibronectin-derived motif that promotes cell adhesion, either alone or in combination with a MIP, which led to improved cell binding (Lauria et al., 2017). In these studies, the mineralization capability was comparable among different peptide modifications, and scanning electron microscopy coupled with energy dispersive X-ray spectroscopy confirmed the presence of calcium and phosphate. Recombinant PVX particles embedded in agarose hydrogels served as biomimetic nanocomposites building up filamentous and network-like nanostructures and stimulating osteogenic differentiation *in vitro* in human bone marrow-derived mesenchymal stromal cells. For tissue engineering applications, it is not only important to control the size and shape of hydroxyapatite crystals but also to ensure the scaffold is compatible with the cells (Klein et al., 1994). Mineralized recombinant PVX particles may therefore be useful in bone tissue engineering, regeneration and restoration by mimicking certain aspects of the bone extracellular matrix. PVX-MIP particles offer a promising biomimetic composite for the synthesis of such bone-like materials.

### Knowing Me, Knowing You: Biosensing

Potato virus X has been fused to the B domain of *Staphylococcus aureus* Protein A to achieve efficient antibody capture and presentation on the particle surface. Protein A binds to the Fc region of immunoglobulins (especially IgG) from many species, and is therefore routinely used for antibody purification and immunoprecipitation. The Protein A fragment retained its ability to immobilize antibodies when exposed on the PVX surface as a direct CP fusion (Uhde-Holzem et al., 2016). The particles were then immobilized on gold chips and used for quartz crystal microbalance detection. The modified PVX particles were able to capture 300–500 antibodies per particle, which enhanced the available antibodies on the chip surface and allowed the sensitive detection of Cowpea mosaic virus. In addition to sensing applications, the arrays could be used in the future to capture pollutants for cleanup or detoxification. Furthermore, when combined with medical payloads, such as contrast agents or drugs, the particles could be used for molecular imaging and drug delivery.

Potato virus X has also been used to improve an ELISA for the diagnosis of primary Sjögren Syndrome (Tinazzi et al., 2015). PVX was genetically modified to display the immunodominant lipo-peptide from lipocalin, which is involved in the pathogenesis of this autoimmune disease. The modified particles were used to coat ELISA plates for the analysis of patient serum samples and were compared to plates coated with the lipocalin peptide alone. The new ELISA achieved a sensitivity of 86.8% for the synthetic peptide but 98.8% for the PVX-lipocalin particles, a remarkable improvement. The ELISA plates could be stored for 60 days with no loss of diagnostic sensitivity.

Potato virus X has also been engineered for the selective attachment of target proteins or molecules via non-covalent interactions. A hybrid in which 50% of the CPs were fused to a single-chain antibody was created using the FMDV 2A sequence 20 years ago (Smolenska et al., 1998). Among the many potential applications of this platform, the authors produced a single chain antibody against the herbicide 3-(3,4-dichlorophenyl)-1,1-dimethylurea, and proposed that the PVX-antibody particles would be suitable for the remediation of contaminated soil and waterways. However, the recombinant virus remained infective, so careful precautions would be necessary before releasing it into the environment.

### A Little More Action, Please: Catalysis

Many industrial processes require enzymes or enzyme cascades that survive harsh process conditions such as high temperatures or extreme pH for efficient substrate conversion. Additionally, the enzymes should be stable and reusable to prolong their retention and minimize process costs. The latter can be achieved by immobilization on solid supports, which in some cases even improves the enzyme stability (Rodrigues et al., 2013).

With its high aspect ratio, PVX is an ideal scaffold for the presentation of multiple copies of small peptides and proteins, including enzymes. Despite the constraints of genetic modification, this is the preferred method to develop new PVX-based biocatalysts because large quantities can be produced by molecular farming within 2–3 weeks (see Don’t Stop ‘Til You Get Enough: Production of VNPs). Carette et al. (2007) took advantage of this one-step production system to create stable PVX nanoparticles presenting a commercially important lipase from *Candida antarctica* (CALB). To circumvent the size limitation during particle assembly, they used the overcoat strategy to produce hybrid particles containing recombinant and wild-type CPs. CALB retained its activity when immobilized on the particle surface, as shown by *in situ* single enzyme studies with the profluorescent substrate 5(6)-carboxyfluorescein diacetate. However, the catalytic activity of the hybrid virus particle against the substrate *p*-nitrophenyl caproate was 2 μmol min^-1^ mg^-1^. This is 45 times lower than the free enzyme, possibly because the CP fusion hindered substrate access to the active site. As stated earlier, the overcoat strategy is also incompatible with enzymes that require post-translational modification, such as the predominantly glycosylated enzymes from *Trichoderma reesei*. Klose et al. (2015) demonstrated that the expression and activity of these enzymes is also dependent on subcellular targeting, so a site-specific attachment system is desirable. One example is ST/SC (see Catch Me If You Can: Sticky Particles), which is based on the CnaB2 domains of the fibronectin-binding protein of *Streptococcus pyogenes* (Zakeri et al., 2012). These components rapidly form an irreversible and specific covalent bond, allowing for positional control across a broad range of buffers and temperatures. We recently engineered PVX nanoparticles displaying the shorter ST and expressed the *T. reesei* endoglucase Cel12A-SC fusion protein in a different plant cell compartment to facilitate glycosylation (Röder et al., 2017). This system achieved a three-fold higher coupling efficiency than the CALB overcoat strategy even though the lipase is smaller than Cel12A-SC. The resulting VNP displayed ∼850 enzymes per PVX particle, and the retention of catalytic activity was confirmed by measuring kinetic parameters in the presence of different concentrations of 4-methylumbelliferyl-β-D-cellobioside. The affinity of the PVX-ST/Cel12A-SC nanoparticles for the substrate was ∼3.5-fold lower than the free enzyme, indicating that the scaffold interferes with substrate binding to some degree. This issue could be addressed by adding a carbohydrate binding module to anchor the cellulose chains. However, the turnover rate k_cat_ and V_max_ of the PVX-ST/Cel12A-SC particles was ∼2.9-fold higher than the free enzyme, which may reflect ability of closely spaced enzymes on the 515-nm scaffold to facilitate hydrolysis.

Cellulose can be broken down into single glucose molecules by the synergistic activity of exoglucanases, endoglucanases and β-glucosidases. Using the ST/SC approach, a system has been designed to allow the future development of stoichiometric multi-enzyme cascades immobilized on PVX VNPs. Moreover, the ST-engineered PVX is a promising universal platform for the attachment of single or multiple proteins that cannot be fused to the CP by genetic engineering.

## How Far We’ve Come: Perspectives

This article highlights the great potential of PVX as a platform for the development of novel VNPs. However, certain challenges remain to be overcome. One limitation for the commercial development of PVX and plant viruses in general is the purification process. The current purification protocol involves several ultracentrifugation steps (see Don’t Stop ‘Til You Get Enough: Production of VNPs) which are unsuitable for large-scale processes. A scalable purification protocol which might also be compatible with good manufacturing practices therefore needs to be established. We have also observed that the particle yield *in planta* and throughout the purification process depends on the peptide fusion. During the first steps of the purification process, the precipitation steps can easily be improved by adjusting the pH of the extraction buffer according to the pI of the CP fusion protein. We found that peptide fusions with lower pI values often cause severe infections in host plants but produce fewer particles (Lauria et al., 2017). Peptide fusions near or slightly higher than the pI of the wild-type CP most likely produce higher particle yields. Although CP protein fusion via the 2A sequence can enlarge the spectrum of potential fusion partners, the yields *in planta* and after purification are even lower than those achieved using the direct fusion strategy. Moreover, CP fusions tend to become less stable at the genomic and protein levels as the insert size increases. The use of different 2A sequences with different processivities may improve the stability and optimize the ratio of fusion protein to free CP on the particle surface.

The limitations of the VNP approach include the tendency of RNA viruses to delete non-essential foreign sequences and their incompatibility with target proteins that require post-translational modification. One possible solution is the use of ST/SC chemistry to covalently attach proteins that are not encoded in the virus genome and that can be modified post-translationally in other subcellular compartments before attachment, a strategy that also increased the coupling efficiency by about three-fold compared to the FMDV 2A approach (Röder et al., 2017). By using this configuration, it might be possible to stably immobilize entire enzyme cascades on a single PVX scaffold, thus taking advantage of proximity effects. To control the enzyme stoichiometry, sticky proteins with different coupling efficiencies can be combined, such as ST/KTag/SpyLigase or SnoopTag/SnoopCatcher (Fierer et al., 2014; Siegmund et al., 2016; Veggiani et al., 2016; Brune et al., 2017; Bao et al., 2018; van den Berg van Saparoea et al., 2018). These new fusion strategies are currently only possible using small batch production methods and it will be necessary to develop innovative approaches to increase the production scale. However, as these sophisticated approaches become more widespread, we will see an ever increasing spectrum of potential applications for engineered PVX particles, spanning the fields of medicine, biomedical engineering, material science and industrial biocatalysts.

## Author Contributions

JR and CD contributed equally to this work. All authors read and approved the final manuscript.

## Conflict of Interest Statement

The authors declare that the research was conducted in the absence of any commercial or financial relationships that could be construed as a potential conflict of interest.

## Abbreviations

CALB: *Candida antarctica* lipase B
CP: coat protein
dpi: days post-inoculation
ELISA: enzyme-linked immunosorbent assay
FMDV: Foot-and-mouth disease virus
GFP: green fluorescent protein
MIP: mineralization-inducing peptide
ORF: open reading frame
pI: isoelectric point
PEG: polyethylene glycol
PVX: Potato virus X
SC: SpyCatcher
sgRNA: subgenomic RNA
ST: SpyTag
TGB: triple gene block
TMV: Tobacco mosaic virus
VNP: virus nanoparticle
